# Prostaglandin E2 Enhances Gap Junctional Intercellular Communication in Clonal Epithelial Cells

**DOI:** 10.3390/ijms22115813

**Published:** 2021-05-28

**Authors:** Alejandro Ogazon del Toro, Lidia Jimenez, Mauricio Serrano Rubi, Aida Castillo, Lorena Hinojosa, Jacqueline Martinez Rendon, Marcelino Cereijido, Arturo Ponce

**Affiliations:** Department of Physiology, Biophysics and Neurosciences, CINVESTAV-IPN, CDMX, México C.P. 07360, Mexico; aogazon@cinvestav.mx (A.O.d.T.); lidiajp@webmail.fisio.cinvestav.mx (L.J.); mauricio.serrano@cinvestav.mx (M.S.R.); aida.castillo@cinvestav.mx (A.C.); lorena.hinojosa@cinvestav.mx (L.H.); jmrendon@cinvestav.mx (J.M.R.); cereijido@cinvestav.mx (M.C.)

**Keywords:** prostaglandin E2, gap junction, dye transfer, epithelia

## Abstract

Prostaglandins are a group of lipids that produce diverse physiological and pathological effects. Among them, prostaglandin E2 (PGE2) stands out for the wide variety of functions in which it participates. To date, there is little information about the influence of PGE2 on gap junctional intercellular communication (GJIC) in any type of tissue, including epithelia. In this work, we set out to determine whether PGE2 influences GJIC in epithelial cells (MDCK cells). To this end, we performed dye (Lucifer yellow) transfer assays to compare GJIC of MDCK cells treated with PGE2 and untreated cells. Our results indicated that (1) PGE2 induces a statistically significant increase in GJIC from 100 nM and from 15 min after its addition to the medium, (2) such effect does not require the synthesis of new mRNA or proteins subunits but rather trafficking of subunits already synthesized, and (3) such effect is mediated by the E2 receptor, which, in turn, triggers a signaling pathway that includes activation of adenylyl cyclase and protein kinase A (PKA). These results widen the knowledge regarding modulation of gap junctional intercellular communication by prostaglandins.

## 1. Introduction

Prostaglandins (PGs) are a group of lipid compounds that share similar properties, both in their chemical structure and in their function. PGs are a class member of the family of eicosanoids, which also include leukotrienes and thromboxanes [1]. PGs are produced enzymatically, by cyclo-oxygenases (COXs), and from arachidonic acid, a 20-carbon polyunsaturated, essential fatty acid [2,3]. These compounds are produced by almost every cell of animal species. In most cases, they act as autocrine and paracrine hormone-like mediators (i.e., they signal at or immediately adjacent to their site of synthesis) [4,5]. Playing a role as signaling starters of diverse signaling pathways, PGs are involved in a variety of effects in numerous physiological and pathological processes [6,7], among which inflammation stands out [8,9] but which also include cancer [10,11], heart disease [12,13], labor and delivery [14,15,16], hypertension [17,18], asthma and allergic diseases [19], migraine [20], sleep [21], bone metabolism [22,23], rheumatoid arthritis [24], and blood flow and the formation of blood clots [25], among many others.

Among the distinct types of prostaglandins, prostaglandin E2 (PGE2), also known as dinoprostone, is perhaps the most important because it is the most abundant in the human body [26,27], as well as because of the wide variety of functions in which it participates, including fever, kidney function, pain, mucosal integrity, blood pressure regulation, and inflammation. PGE2 exerts its actions by acting on a group of G-protein-coupled receptors (GPCRs). There are four GPCRs responding to PGE2-designated subtypes EP1, EP2, EP3, and EP4. The EP subtypes exhibit differences in signal transduction, tissue localization, and regulation of expression [28,29].

Gap junctions are molecular structures that, as channels, communicate two neighboring cells through the exchange of ions and molecules of low molecular weight. Each gap junction channel is formed by two connexons or hemichannels contributed by each communicating cell. A connexon is an assembly of six proteins subunits called connexins, forming a pore [30,31,32,33,34]. Gap junctions are expressed in a wide variety of animal tissues; some notable examples are the cardiac muscle [35,36], smooth muscle [37,38], and neurons [39,40], allowing electrical communication. In endocrine glands, gap junctions help synchronize the release of hormones in response to a given stimulus [41,42]. In epithelia, they participate significantly in the processes of differentiation or modulation of trans-epithelial transport [43,44].

Although knowledge about the variety and functions of prostaglandins has advanced notoriously in the past decades, so far, there are only a few examples describing a role of these compounds in gap junctions. In human lung (WI-38) cells, prostaglandin E1 induces the upregulation of junctional membrane permeability and junctional membrane particles [45]; in the rat epididymis, PGE2 activates signaling pathways implicated in the expression and phosphorylation of connexin 43 [46]; in the rat myometrium, steroid hormones and prostaglandins interact to modulate gap junction formation [47]; and in osteocyte-like MLO-Y4 cells, PGE2 modulates gap junctional intercellular communication (GJIC) induced by mechanical strain [48].

In this work, we set out to determine whether PGE2 influences gap-junction-mediated intercellular communication in renal epithelial cells (MDCK cells). For this purpose, we performed dye transfer assays at different times and concentrations. We also discerned which receptor mediates the effect of PGE2 and tested whether protein kinase A (PKA) and cAMP are involved downstream in the signal pathway.

## 2. Results

### 2.1. PGE2 Induces Enhancement of Gap Junctional Intercellular Communication in Epithelial Cells

**Figure 1:**
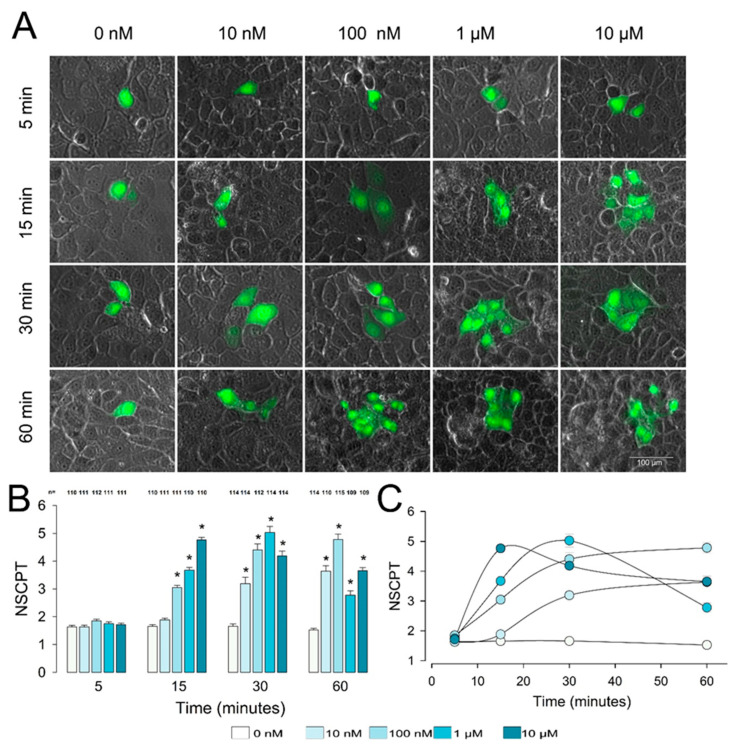
Prostaglandin E2 (PGE2) induces gap junctional intercellular communication in epithelial cells. (**A**). Representative images show clusters of cells stained (green) after one of them was injected with Lucifer yellow. Images were selected to match the median of the number of stained cells per trial (NSCPT) under each experimental condition, as indicated. (**B**) The bar chart compares the average (NSCPT) of the distinct concentrations of PGE2 versus the control (0 nM) at each time tested. The * above bars indicate a statistically significant difference of a given concentration of PGE2 versus the control. The numbers above each bar indicate the number of repeats under each experimental condition. (**C**) A plot of NSCPT vs. time shows the kinetic of the response at each experimental concentration of PGE2. The continuous lines were drawn by smoothing.

To determine whether PGE2 induces GJIC in epithelial cells, we performed dye transfer assays (as detailed in the Materials and Methods ection) at different concentrations (10 nM, 100 nM, 1 µM, and 10 µM) and at different times (5, 15, 30, and 60 min). We considered the number of cells stained as a result of having injected a single cell (number of stained cells per trial (NSCPT)) as an estimate of GJIC. For each experimental group (time, concentration), we made a determined number of trials to calculate its average value (mNSCPT) and the standard error of the mean (SE). Each repeat was obtained by injecting a single cell selected at random from those constituting an epithelial monolayer. Figure 1A shows a series of representative images, one for each experimental condition, while part (B) is a bar chart comparing, at each time of treatment, the mNSCPT (±SE) of the different concentrations of PGE2 against their respective control.

After 5 min of treatment, PGE2 produced no effect, as mNSCPT was not statistically different from the control at any of the concentrations tested. Nonetheless, after 15 min of treatment, PGE2 induced enhancement of the NSCPT, which was statistically significant from 100 nM onward; at this time, mNSCPT increased steadily, without reaching a maximum. After 30 min of treatment, PGE2 produced a statistically significant increase, compared to the control, from 10 nM onward. mNSCPT also increased with concentration, up to a maximum at 1 µM, and then decreased slightly at 10 µM. After 60 min, the maximum value of mNSCPT was produced by 100 nM of PGE2. With 1 µM, this value decreased slightly, and then increased again with 10 µM. Figure 1C is a plot showing mNSCPT (±SE) versus time for each of the concentrations tested. It is interesting that at lower concentrations (10 and 100 nM), mNSCPT rose to a steady value, whereas at 1 and 10 µM, it reached a peak and then decreased.

These results show that PGE2 induces a dose-dependent increase in GJIC in MDCK epithelial cells. From the data obtained, we calculated an IC50 of 5.19 nm when the cells are treated for 30 min.

### 2.2. Effect of Gap Junction Blockers on PGE2-Induced GJIC Enhancement

Next, we tested the effect of a group of four compounds that have been described as gap junction blockers. These tests were done, on the one hand, to show that neighboring cells are effectively stained by diffusion of the dye through gap junctions and, on the other hand, to learn about the molecular nature of the connexins involved in the phenomenon. These blockers were (1) octanol, which in a range of 10–1000 µm blocks GJ formed by Cx43, Cx46, or Cx50 [49,50]; (2) heptanol (1–3 mM), which blocks Cx43, Cx46, and Cx50 [50,51]; (3) niflumic acid (200–500 um), which blocks Cx43, Cx46, and Cx50 [50,52]; and (4) quinine (30–300 µm), which blocks Cx36, Cx45, and Cx50 [50,53].

**Figure 2:**
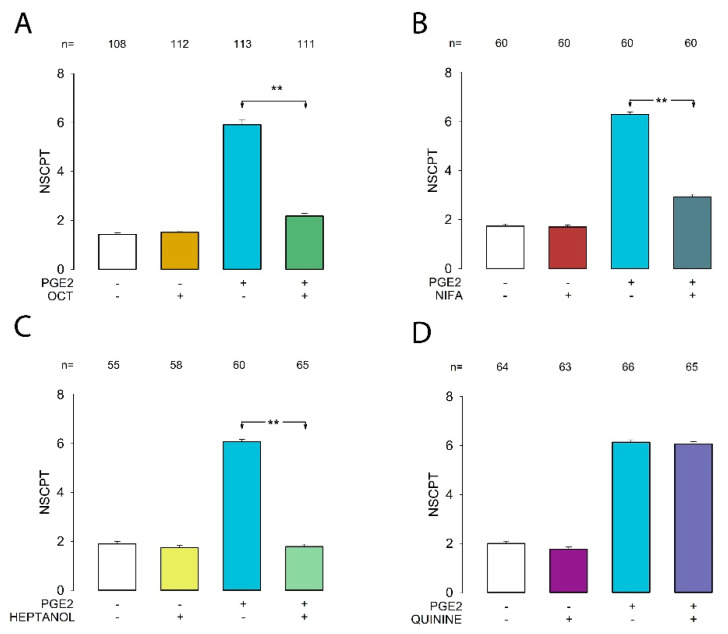
Effect of gap junction blockers on the PGE2-induced enhancement of gap junctional intercellular communication (GJIC). Bar charts (**A**–**D**) show the effect of octanol (OCT), niflumic acid (NIFA), heptanol, and quinine, respectively. Bars show the mNSCPT (±standard error of the mean (SE)) under each experimental condition indicated at the bottom. The ** above bars indicate a statistically significant difference (*p* < 0.001) between the groups indicated by the bars. The numbers above each bar indicate the number of repeats under each experimental condition.

To test the effect of each compound, we performed dye transfer tests to estimate the mNSCPT and SE of the following groups: control, treated with only PGE, treated with only blocker, or treated with PGE2+blocker. The test concentrations were 1 mM, 2 mM, 500 µM, and 300 µM for octanol, heptanol, niflumic acid, and quinine, respectively. As shown in Figure 2, we found that octanol (Figure 2A) and heptanol (Figure 2C) completely blocked the effect, while niflumic acid (Figure 2B) partially blocked it, and quinine (Figure 2D) did not produce a statistically significant effect.

Therefore, these results allow us to confirm that the observed effect is effectively due to modulation of gap junctions. Additionally, they allow us to suggest that gap junctions could be made up of either Cx43, Cx46, or Cx50.

### 2.3. PGE2-Induced Enhancement of GJIC Does Not Involve Synthesis of New Subunits

This PGE2-induced GJIC enhancement may be due to the synthesis of new gap components, the relocation of components already synthesized, or modulation of the activation properties of gap junctions already assembled in the membrane. To evaluate the possibility that new gap junctions are required, we tested the effect of actinomycin D (AD) and cycloheximide (CX) on the response already described.

Actinomycin D is a transcription inhibitor that intercalates into DNA to form a stable complex with DNA, preventing unwinding of the DNA double helix, thus inhibiting the DNA-dependent RNA polymerase activity. Actinomycin D has been widely used in mRNA stability assays to inhibit the synthesis of new mRNA [54,55,56]. It has been proved to be effective at 10 µg/mL [57].

Cycloheximide is a glutarimide antibiotic produced by *Streptomyces griseus* that inhibits protein synthesis in eukaryotes (IC50 = 5–50 μM) by interfering with translational elongation. It is widely used as a tool in molecular biology research for ribosomal profiling and translational profiling as well as for determining the half-life of a protein [58,59,60,61].

To test the effect of AD (10 µg/mL), we performed dye transfer trials and counted the NSCPT under control conditions or with PGE2, AD, or PGE2+AD treatment. To assay the effect of CX (100 µM), we performed dye transfer trials and counted the NSCPT under control conditions or with PGE2, or CX or PGE2+CX treatment.

**Figure 3:**
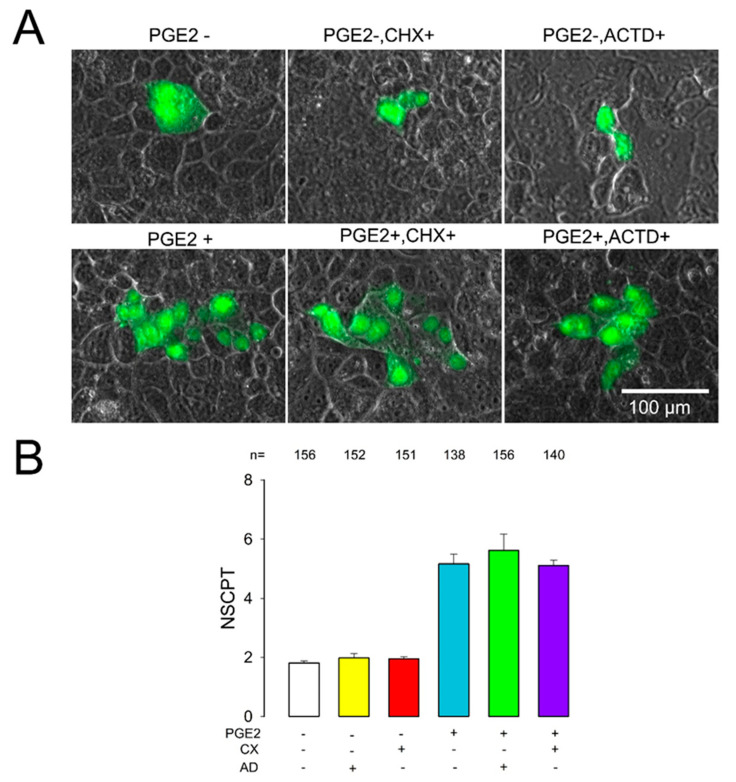
PGE2-induced enhancement of GJIC does not involve synthesis of new subunits. (**A**) Representative images show clusters of cells stained (green) after one of them had been injected with Lucifer yellow. Each image was selected to match the median of the NSCPT obtained from a number of repeats under each experimental condition indicated. (**B**) The bar chart represents the average (NSCPT) under each experimental condition indicated at the bottom. Comparisons of PGE2 vs. PGE2+AD and PGE2 vs. PGE2+CX were not statistically significant. The numbers above each bar indicate the number of repeats under each experimental condition.

As shown in Figure 3, treatment with only AD did not produce a significant difference of mNSCPT compared to the control (1.98 ± 0.15 vs. 1.81 ± 0.07, *p* = 0.31). In addition, the mNSCPT of the group treated with PGE2±AD was not significantly different from that of the group treated with PGE2 (5.75 ± 0.56 vs. 5.16 ± 0.32, *p* = 0.18). Similarly, the mNSCPT of the group treated with only CX was no different from that of the control group (1.76 ± 0.05 vs. 1.81 ± 0.07, *p* = 0.24); likewise, the mNSCPT of the group treated with PGE2+CX was no different from that of the group treated with only PGE2 (5.15 ± 0.17 vs. 5.16 ± 0.32, *p* = 0.29).

These results suggest, therefore, that no synthesis of new mRNA or proteins is required for PGE2 to induce GJIC enhancement.

### 2.4. PGE2 Promotes Transport and Relocation of Gap Junction Components

Next, we tested the hypothesis that PGE promotes relocation of subunits already synthesized toward the membrane and that the participation of traffic mechanisms is required. For this reason, we evaluated the effect of two compounds: brefeldin A and nocodazole.

**Figure 4:**
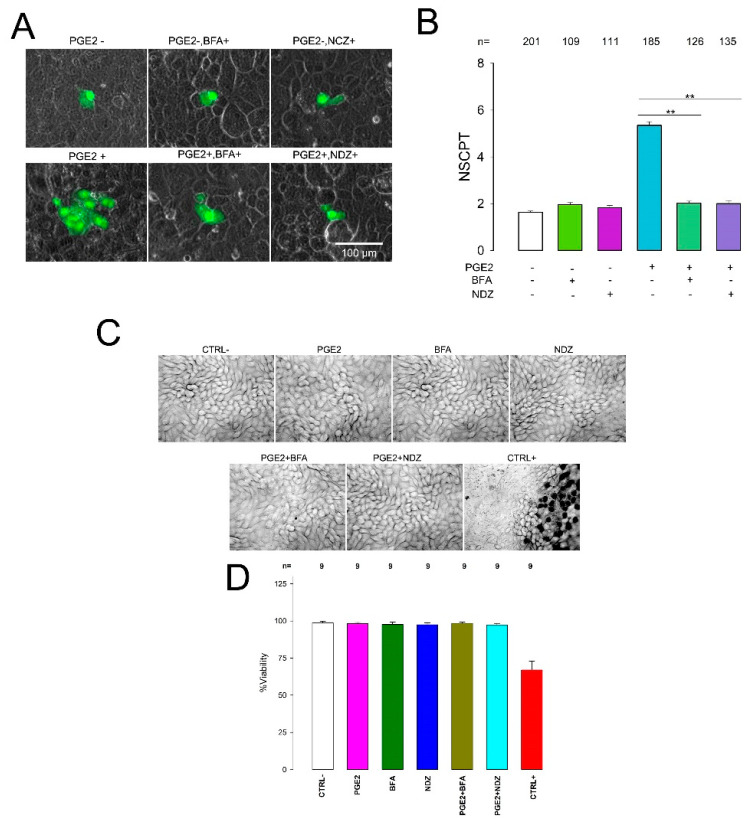
PGE2 promotes transport and relocation of gap junction components. (**A**) Representative images show clusters of cells stained (green) after one of them had been injected with Lucifer yellow. Each image was selected to match the median of the NSCPT obtained from a number of repeats under each experimental condition indicated. (**B**) The bar chart compares the mean NSCPT(±SE) under each experimental condition indicated at the bottom of the bars. (**C**) Representative bright-field images and (**D**) bar chart comparing viability, by Trypan blue staining, of the different experimental conditions, including treatment with brefeldin A (BFA) and nocodazole (NDZ). ** Statistically significant difference (*p* < 0.001) among the bars indicated by the lines. The numbers above bars indicate the number of repeats.

Brefeldin A (BFA) is a natural fungal metabolite that reversibly inhibits vesicle formation and transport between the endoplasmic reticulum and the Golgi apparatus [62,63]. To assay the effect of BFA, we performed dye transfer assays to measure the NSCPT under control conditions or with PGE2, BFA (10 µM), or PGE2+ BFA treatment. As Figure 4 shows, addition of this compound abolished the enhancement caused by PGE2 on GJIC, as the mNSCPT of cells treated with PGE2+BFA was significantly lower than that of cells treated with only PGE2 (1.98 ± 0.15 vs. 5.34 ± 0.15, *p* < 0.001). Treatment of cells with only BFA did not affect GJIC, as the mNSCPT of this group was not statistically different from that of the control (2.01 ± 0.09 vs. 1.82 ± 0.05, *p* = 0.14).

Nocodazole (NDZ) is an antimitotic agent that disrupts microtubules by binding to β-tubulin and preventing the formation of one of the two interchain disulfide linkages, thus inhibiting microtubule dynamics [64,65]. To assay the effect of NDZ, we performed dye transfer assays and counted the NSCPT under control conditions or with PGE2, NDZ (10 µM), or PGE2+NDZ treatment. Figure 4 shows that NDZ also suppressed the enhancement of GJIC, as the mNSCPT of cells treated with PGE2+NDZ was significantly lower than that of cells treated with only PGE2 (2.0 ± 0.13 vs. 5.34 ± 0.15, *p* < 0.001), while treatment of cells with only NDZ did not affect GJIC, as the mNSCPT of this group was not statistically different from that of the control (1.82 ± 0.10 vs. 1.82 ± 0.05, *p* = 0.64). To verify that treatment with inhibitors does not cause significant cell damage, we performed viability tests with Trypan blue staining. As shown in Figure 4C,D), none of the inhibitors produced a significant decrease in viability.

These results then led us to suggest that PGE2 induces an increase in GJIC, promoting the mobilization of subunits, components of gap junctions already synthetized, through mechanisms that depend on the organization of the tubulin cytoskeleton.

### 2.5. Prostaglandin E2 Enhances GJIC via the EP2 Receptor

Our next goal was to find out which receptor mediates this response since, as it is known, PGE2 could exert its effects through any of four separate G-protein-coupled receptors (EP1, EP2, EP3, or EP4) [66,67,68]. For this purpose, we tested the effect of several compounds known to be agonists (butaprost and CAY10399) and antagonists (AH-6809, L-798106, TG4155, and PF-04418948) of EP receptors.

**Figure 5:**
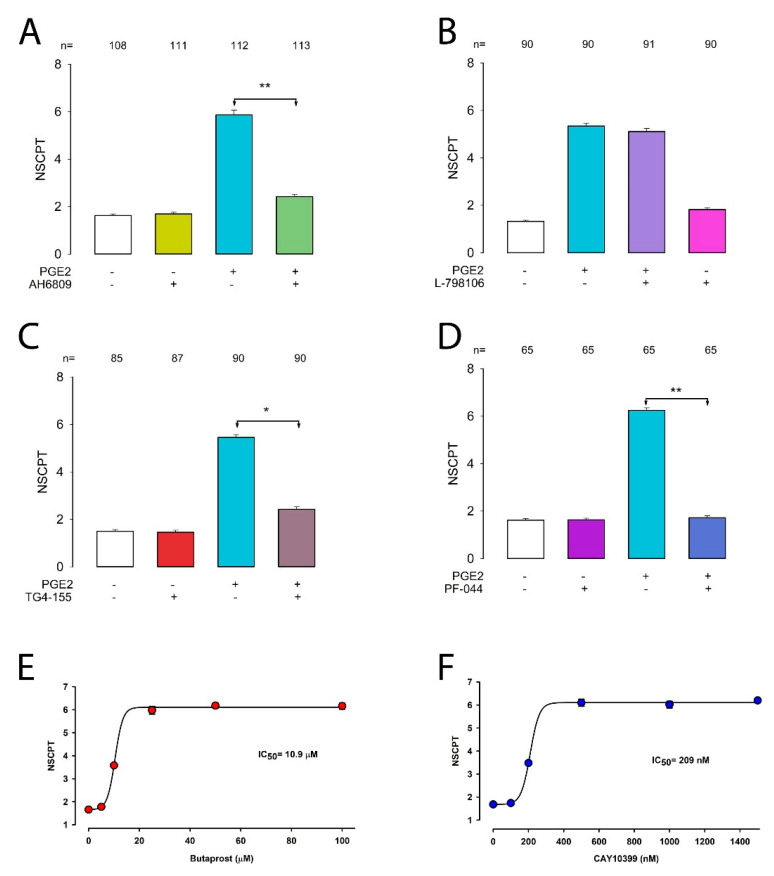
Prostaglandin E2 enhances GJIC via the EP2 receptor. (**A**–**D**) The effect of EP receptor antagonists. Bar charts compare the mNSCPT (±SE) of the given experimental condition indicated below each bar. The numbers above bars indicate the number of repeats. The * and ** symbols indicate a statistically significant difference (p < 0.01) and (*p* < 0.001) respectively between the bars, indicated by the lines. (**E**,**F**) Dose–effect plots show the effect of EP2-selective agonists butaprost and CAY10399, respectively. The continuous lines were calculated from fitting data to a sigmoidal 4-parameter model to obtain IC50.

We first tested the effect of AH-6809, a receptor antagonist with nearly equal affinity for EP1, EP2, and EP3 [69]. To assay the effect of AH-6809, we performed dye transfer assays and counted the NSCPT under control conditions or with PGE2, AH-6809 (100 nM), or PGE2+AH-6809 treatment. As shown in Figure 5A, AH-6809 itself did not produce any effect on GJIC, as the mNSCPT of cells treated with this compound was not statistically distinct from that of the control (1.69 ± 0.15 vs. 1.62 ± 0.06, *p* = 0.43); however, it effectively abolished the enhancement induced by treatment with PGE2, as the mNSCPT of the group treated with PGE2+AH-6809 was significantly lower than that of the group treated with PGE2 (5.86 ± 0.19 vs. 2.42 ± 0.09, *p* < 0.001). These results suggest that PGE2 induces enhancement of GJIC through EP1, EP2, or EP3. Thus, to examine in more detail which of these receptors participates, we used more selective inhibitors. L-798106 is a highly selective EP3 receptor antagonist [70]. To assay the effect of L-798106, we performed dye transfer assays and counted the NSCPT under control conditions or with PGE2, L-798106 (1 nM), or PGE2+L-798106 treatment. As shown in Figure 5B, L-798106 itself did not produce any effect on GJIC, as the mNSCPT of cells treated with this compound was not statistically distinct from that of the control (1.61 ± 0.06 vs. 1.35 ± 0.07, *p* = 0.03). However, this compound did not abolish the enhancement of GJIC induced by treatment with PGE2, as the mNSCPT of the group treated with PGE2+L-798106 was not significantly different from that of the group treated with only PGE2 (5.11 ± 0.34 vs. 5.34 ± 0.11, *p* = 0.19).

The results obtained in this assay indicate that EP3 is not involved, therefore, suggesting, that either EP1 or EP2 could be the receptor that participates in the effect that PGE2 causes on GJIC. To further elucidate this hypothesis, we test TG4155, a highly selective EP2 antagonist, with Ki of 2.4 nM [71,72]. To assay the effect of TG4155, we performed dye transfer assays to estimate mNSCPT under controlled conditions or with treatment of TG4155 (5 nM) only, or with PGE2, or PGE2+ TG4155. As shown in Figure 5C, TG4155 itself did not produce any effect on GJIC, as mNSCPT of cells treated with this compound was not statistically distinct of that of control (1.69 ± 0.15 vs. 1.62 ± 0.06, *p* = 0.43); however, it effectively abolished the enhancement induced by treatment with PGE2, as mNSCPT of the group treated with PGE2+ TG4155 was significantly lower than the group treated with only PGE2 (2.42 ± 0.10 vs. 5.45 ± 0.11, *p* < 0.001).

These results therefore suggest that EP2 and not EP1 is the receptor involved in PGE2-induced GJIC enhancement. To support further this hypothesis, we assayed the effect of another antagonist (PF-04418948) and two EP2-selective agonists (butaprost and CAY10399).

PF-04418948 is a potent and selective EP2 receptor antagonist (IC50 = 16 nM), which is over a 1000-fold less active than other EP receptors [73]. To assay the effect of PF-04418948, we performed dye transfer assays to estimate the mNSCPT under control conditions or with PF-04418948 (50 nM), PGE2, or PGE2+PF-04418948 treatment. As shown in Figure 5D, PF-04418948 itself did not produce any effect on GJIC, as the mNSCPT of cells treated with this compound was not statistically distinct from that of the control (1.63 ± 0.07 vs. 1.62 ± 0.07, *p* = 0.52); however, it effectively abolished the enhancement induced by treatment with PGE2, as the mNSCPT of the group treated with PGE2+PF-04418948 was significantly lower than that of the group treated with only PGE2 (1.72 ± 0.08 vs. 6.24 ± 0.11, *p* < 0.001).

Butaprost, a structural analog of PGE2, is a selective agonist for the EP2 receptor subtype, which binds to the EP2 receptor with an IC50 of 5 µM and does not bind appreciably to any of the other EP receptors. This compound has frequently been used to pharmacologically define the EP receptor expression profile of various human and animal tissues and cells [74,75]. We assayed the effect of butaprost, measuring the NSCPT at several concentrations, ranging from 0 to 100 µM (Figure 5E). The value of IC50 obtained from fitting the data (10.4 µM) was roughly comparable to the IC50 (5 µM) described for EP2 before.

CAY10399 (the two-series congener of butaprost free acid) is another useful tool for characterizing EP2 receptor-mediated signaling events. It exhibits, as butaprost, a remarkably significantly higher selectivity for EP2 over other EP receptors, although with a greater sensitivity than butaprost (IC50 = 92 nM) [76]. We assayed the effect of CAY10399, measuring the NSCPT at several concentrations, ranging from 0 to 1500 nM (Figure 5F). The value of IC50 obtained from fitting the data was 209 nM, which is comparable to the value of 92 nM reported.

Thus, the results obtained with antagonists and agonists altogether support the hypothesis that EP2 is the receptor type that mediates the PGE2-induced response on GJIC.

### 2.6. Inhibition of cAMP and PKA Diminishes the Effect of PGE2 on CIGJ

It is known that the binding of PGE2 to EP2 activates a G protein, which, in turn, promotes a stimulatory signaling pathway, which includes activation of an adenylyl cyclase (increasing cAMP), as well as activation of PKA [77]. To find out whether these two components also participate in the signaling pathway by which PGE2 induces enhancement of GJIC, we assayed the effect of the inhibitors of each of these two enzymes.

**Figure 6:**
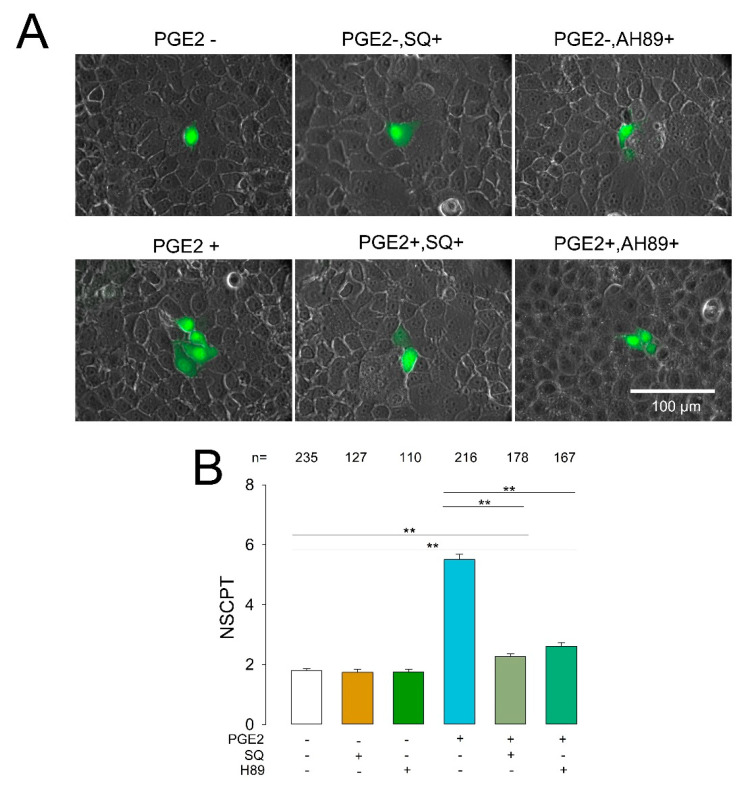
Inhibition of cAMP and protein kinase A (PKA) diminishes the effect of PGE2 on CIGJ. (**A**) Representative images show clusters of cells stained (green) after one of them had been injected with Lucifer yellow. Each image corresponds to the median of the NSCPT obtained from a number of repeats under each experimental condition indicated. (**B**) The bar chart compares the average number of stained cells (NSCPT) under each experimental condition, including controls (untreated cells). The ** above bars indicate a statistically significant difference (*p* < 0.001) between the groups indicated by the lines. The numbers above each bar indicate the number of repeats under each experimental condition.

We tested the effect of SQ22536, which is known to inhibit adenylyl cyclase, thereby blocking the synthesis of cAMP [78]. For this purpose, we performed dye transfer trials to estimate the mNSCPT under control conditions or after treatment with SQ22536 (100 µM), PGE2, or PGE2+SQ22536. As shown in Figure 6, treatment with SQ22536 did not produce any significant effect on GJIC, as the mNSCPT of cells treated with this compound was not statistically different from that of the control (1.74 ± 0.09 vs. 1.80 ± 0.05, *p* = 0.17); however, treatment with PGE2+SQ22536 significantly reduced the mNSCPT as compared to only PGE2 treatment (2.26 ± 0.10 vs. 5.50 ± 0.18, *p* < 0.001).

On the other hand, we tested the effect of H-89, which is known as a potent PKA inhibitor [79,80]. To assay the effect of H-89, we performed dye transfer assays and counted the NSCPT under control conditions or with PGE2 (100 nM, 30 min), H-89 (100 µM), or PGE2+H-89 treatment. Treatment with only H-89 did not produce any significant effect on GJIC, as the mNSCPT of cells treated with this compound was not statistically different from that of the control (2.61 ± 0.12 vs. 1.80 ± 0.05, *p* = 0.32); however, treatment with PGE2+H-89 significantly reduced the mNSCPT as compared to only PGE2 treatment (2.26 ± 0.10 vs. 5.50 ± 0.18, *p* < 0.001).

These results indicate, therefore, that both adenylyl cyclase and PKA are included in the signaling pathway by which PGE2 influences GJIC epithelial cells.

## 3. Discussion

Since their discovery, from seminal fluid in 1930 [81,82,83], prostaglandins have become one of the most widely investigated substances, due in large part to the wide range of physiological and pathological processes in which they participate [84,85]. One proof of their importance is that a wide variety of drugs have been developed around them with widely different purposes, such as the control of inflammation, fever, pain, childbirth pain, rheumatoid arthritis, and blood clotting, starting with aspirin and later with other medications known as nonsteroidal anti-inflammatory drugs (NSAIDs) that, as aspirin does, work by inhibiting the activity of cyclo-oxygenase enzymes (COX-1 or COX-2) [86,87,88]. Nonetheless, although prostaglandins are known to be involved in a wide variety of clinical phenomena and much information has been gathered about the signaling cascades they trigger, some aspects of their effects at the cellular and molecular levels remain poorly described and understood. An example of this fact is the little information available about the effect of prostaglandins on gap junctions, even though the latter are of primary importance in almost any type of animal tissue.

In this work, we investigated whether prostaglandin E2 produces an effect on gap-junction-mediated intercellular communication in MDCK cells, a dog-derived cell line that has classically been used as a model to study epithelial properties [89,90,91,92,93]. The method we used (transfer of Lucifer yellow) is one of the most used methods for GJIC assays [94,95,96], and we have used it in several other studies for various purposes [97,98,99].

As described in detail in the results, we found that PGE2 effectively influences GJIC in MDCK epithelial cells, starting at 10 nM and from 15 min of treatment. Above 10 nM, the degree of communication is significantly increased. It is interesting to point out that according to our observations, the speed with which GJIC increases depends on the concentration, but at concentrations greater than 100 nM, the response reaches a maximum and then decreases, even if treatment with PGE2 is maintained. This might lead to the thought that PGE2 promotes the mobilization of a pool of subunits, already synthesized, toward the membrane, and that such mobilization is faster when the concentration of PGE2 is higher. A subsequent reduction in the degree of GJIC, observed at concentrations greater than 100 nM, could be due to depleted reserves of synthesized subunits. In addition, as already described, we found that treatment for 30 min with either cycloheximide or actinomycin D did not change the effect of PGE2 on GJIC, suggesting that at least at this time, no mRNA or protein synthesis is required, although that does not exclude the possibility that this would happen with larger times.

Regarding the receptors involved in this response, as is already known, PGE2 can influence through four types of receptors (EP1, EP2, EP3, and EP4) [66,67,68]. Other studies have already shown that MDCK cells express the four types of receptors for PGE2 [100]. Our results led us to conclude that EP2 is the receptor that mediates the enhancement of GJIC caused by PGE2, as evidenced by the effect obtained from the antagonists and agonists tested: (1) L-798106, a selective EP3 inhibitor, did not change the response, therefore excluding the involvement of EP3; (2) selective EP2 antagonists TG4155 and PF-04418948; and (3) EP2-selective agonist induced the same response as that of PGE2 with IC50 within the range described in references.

The hypothesis that EP2 is the receptor that mediates the effect of PGE2 is further strengthened by the finding that both PKA and adenylyl cyclase are players in the signaling pathway that leads to enhancement of GJIC. As widely described, the PGE2-EP2 signaling pathway exerts its function through activation of the cyclic AMP–PKA pathway [77,101], and as shown before, treatment with the inhibitors of these enzymes (SQ22536 and H89) significantly suppresses the enhancement of GJIC induced by PGE2. However, as was also shown, the effect was not fully reduced, as the value of the treatment was even significantly greater than the control, suggesting that there is a residual effect that could be due to the participation of another signaling route.

The results obtained with GJIC blockers allow us to confirm that the phenomenon described is effectively due to a modulating action on gap junctions and leads us to suggest that one of Cx43, Cx46, or Cx50 could be the connexin type involved. As in other studies with MDCK cells, we described Cx43 to be expressed in MDCK cells [102], and we could suggest that in this case, Cx43, rather than Cx46 or Cx50, could be the actual connexin type being modulated by PGE2; however, we cannot rule out the participation of other connexins. This needs to be addressed with molecular and immunocytochemical approaches that we plan to pursue in the future.

**Figure 7:**
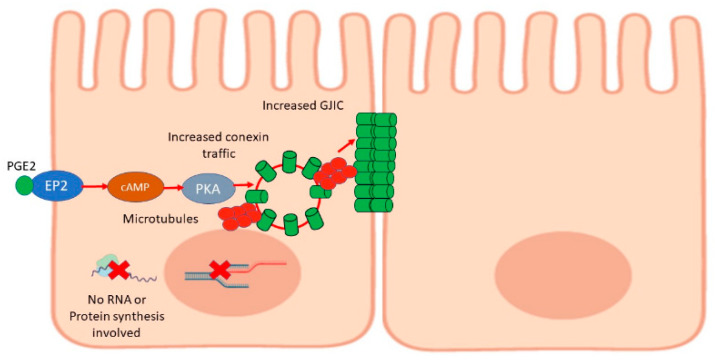
Scheme that summarizes the findings of this work: the binding of PGE2 to the EP2 receptor activates the cAMP and PKA pathway, having the effect of enhancing GJIC. No synthesis of mRNA or proteins is required; however, the effect depends on the vesicular traffic, suggesting that PGE2 stimulates the increase in the traffic of conexins already synthesized.

The results shown in this work, summarized in Figure 7, are somewhat comparable to other works describing the influence of prostaglandins on gap junctions or GJIC. In rat osteoblasts, it has been observed that in addition to inducing a change in their shape, PGE1 and PGE2 induce an increased number of gap junctions (as observed from microscopy), from 1 nM and from 10 min of treatment [103]. In addition, in osteocyte-like cells (MLO-Y4), PGE2 seems to be an essential mediator between mechanical strain and gap junctions. It induced a significant enhancement of GJIC from 1 µM when treated for 16 h [48]. On the other hand, it has been described that in normal endometrial epithelial cells, PGE2 enhances GJIC from 1nM, and higher concentrations decrease the effect, while in human endometrial carcinoma cell lines (FEEC, HEC-1A, and RL-95-2), the optimal PGE2 concentration seems to be higher (1–10 µM). This difference could be attributed to the availability of EP receptors [104]. Apart from these studies, there are no more references that relate PGE2 to GJIC; however, these results are interestingly comparable to ours. The difference in sensitivity of PGE2 could, in the case of MDCK cells, also be attributed to the density of receptors available in the membrane.

Other studies have described that in MDCK cells, prostaglandins prompt diverse actions. For example, it has been observed that PGE1 induces an increase in Na-K-ATPase units via EP1 and EP2 receptors, in both cases increasing cAMP levels [100]. In addition, it has been described that PGE2 influences the growth of MDCK cells, although with antagonic effects, depending on the EP receptor mediating the response. While activation of Gs-coupled EP2 and EP4 by PGE2 results in increased growth, EP1 activation is growth-inhibitory [105].

Up to this point, we do not know whether the observations described here on the PGE2-induced enhancement of GJIC do apply to other tissues in which gap junctions play a crucial role, such as cardiac or endocrine tissue, and whether there is any link between inflammation, in which prostaglandins play a remarkable role, and the GJIC of the affected tissues. Since MDCK cells are of renal origin, these observations may apply only to renal tissues, although it is difficult to speculate on the physiological or pathological consequences at this time. In addition, we do not know how this effect is related to some of the effects that prostaglandins cause in the kidney, regulating renal blood flow as well as ion transport.

Nonetheless, growth, another described effect that prostaglandins have on renal and other tissues, could be related to GJIC. This is an interesting possibility that deserves to be evaluated, because this could set prostaglandins as interesting players influencing the key aspects of cell growth and control, altered in cancer, such as adhesion, migration, and malignity [106].

## 4. Materials and Methods

### 4.1. Cell Culture

Starter MDCK-II cell cultures were obtained from the American Type Culture Collection (MDCK, CCL-34) and maintained in Complete Dulbecco′s Modified Eagle′s Medium (CDMEM) supplemented with 10% fetal bovine serum (FBS) and 10,000 U/mg/mL of penicillin-streptomycin. To make dye transfer assays, cells were trypsinized and seeded at 80% confluence on glass coverslips, placed in 24-well cell culture plates to form mature monolayers, and incubated with CDMEM + 10% FBS. After 24 h, the FBS in the culturing media was reduced to 1% to avoid the possibility of interfering prostaglandins, and the coverslips containing monolayers were kept in this depleted medium for 24 h. After this, the wells containing coverslips were treated with PGE at the concentrations and times described in the Results section, before performing dye transfer trials.

### 4.2. Measurement of Gap Junctional Intercellular Communication by Dye Transfer Assays

Dye transfer assays consisted of impalement and injection, with glass micropipettes, of Lucifer yellow on individual cells from mature monolayers grown on glass coverslips. The coverslips on which cell monolayers had been grown were placed in a translucent chamber filled with PBS plus Ca^2+^ (1.8 mM) solution at room temperature. Micropipettes were elaborated from borosilicate glass capillaries tubes (Kimax, 34500-99) on a vertical David–Kopf puller (DKI-700c). Those with a tip electrical resistance of 5–10 MOhms were backfilled with a saline solution containing 120 mM KCl, 5 mM NaCl, 1 mM MgCl_2_, and 5 mM HEPES, (pH 7.4) and Lucifer yellow (1%). After filling up, the pipettes were attached to the holder device, which was mounted to a micromanipulator (PCS-750; Burleigh Instruments, NY, USA). For impalement of cells, the chamber was mounted on the stage of an inverted microscope (Diaphot 300; Nikon, Tokyo, Japan) equipped with epifluorescence. Three independent trials were made. In each trial, about 30 repeats were performed per coverslip. At each repeat, cells were randomly chosen from those constituting the monolayer and then impaled and injected, one at a time, using a pneumatically driven microinjecting device (IM300; Narishige, NY, USA). After about 30 to 50 cells were injected, the coverslips were rinsed with PBS and fixed by dipping into 4% paraformaldehyde, then rinsed (3×) with PBS, and mounted using VECTASHIELD^®^ (H-1000; Vector Laboratories, Burlingame CA, USA). Eight-bit images of the fluorescent cells were acquired at room temperature using an inverted microscope equipped with a Plan-NeoFluar 63 × N.A. 1.25 objective lens, and AxioCam MRm camera (Zeiss M200; Jena, Germany), and Axovision 4.8 software (www.axovision.com (access on 27 February 2021), Hanover, Germany). The captured images were imported to FIJI Is Just ImageJ software (release 2.8, NIH, Bethesda, MD, USA) to adjust the brightness and the contrast and GIMP (release 2.8.10, NIH) to compose the figures.

### 4.3. Chemicals

All chemicals and reagents were obtained from Sigma. TG4155 (Cat SML1217) was dissolved in DMSO at 10 mg/mL and diluted to a working concentration of 5 nM, L-798106 (Cat L4545) was dissolved in DMSO at 15 mg/mL and diluted to a working concentration of 1 nM, and AH-6809 (Cat A1221) was dissolved in DMSO at 1 mg mL and diluted to a working concentration of 700 nM. Prostaglandin E2 (cat P-5640) was prepared as a 5 g/mL stock in absolute ethanol; subsequent dilutions were prepared in PBS. Lucifer yellow was obtained from Sigma-Aldrich (67764-47-5) and dissolved in a solution containing 100 mM KCl, 5 mM NaCl, 10 mM HEPES, and 1 mM CaCl_2_ (pH 7.4).

### 4.4. Statistical Analyses

The data collected in this work were processed and analyzed statistically using the Microsoft Office 365 Excel and Sigmaplot 12.5. The data were generated by counting the number of cells stained with Lucifer yellow resulting from the injection of a single cell. The results shown are the product of three independent experimental trials. The number of data are indicated in the figures and in the text. The data are represented as the average value and dispersion as the standard error of the mean (SE). Statistical analysis, as indicated in the text and figures, consisted of Analyses of Variance (ANOVA) test, followed by multiple-comparison tests (Holm–Bonferroni method). If the data did not meet the normality criterion (Shapiro–Wilk test) required for parametric analysis, Kruskal–Wallis one-way analysis of variance on ranks was used instead, followed by nonparametric multiple comparisons (Dunn method). Simple paired comparisons were made, either with a t-test or the Mann–Whitney rank sum test. A minimum criterion of *p* < 0.05 was considered for a statistically significant difference.

## Data Availability

Not applicable.

## References

[1] Calder P.C. (2020). Eicosanoids. Essays Biochem..

[2] Oesterling T.O., Morozowich W., Roseman T.J. (1972). Prostaglandins. J. Pharm. Sci..

[3] Samuelsson B., Granstrom E., Green K., Hamberg M., Hammarstrom S. (1975). Prostaglandins. Annu. Rev. Biochem..

[4] Funk C.D. (2001). Prostaglandins and Leukotrienes: Advances in Eicosanoid Biology. Science.

[5] Shimizu N., Nakamura T. (1985). Prostaglandins as hormones. Dig. Dis. Sci..

[6] Poyser N.L. (1973). The physiology of prostaglandins. Clin. Endocrinol. Metab..

[7] Jones R.L. (1972). Functions of prostaglandins. Pathobiol. Annu..

[8] Ricciotti E., FitzGerald G.A. (2011). Prostaglandins and inflammation. Arterioscler. Thromb. Vasc. Biol..

[9] Aoki T., Narumiya S. (2012). Prostaglandins and chronic inflammation. Trends Pharmacol. Sci..

[10] Wang D. (2006). Prostaglandins and cancer. Gut.

[11] Fischer S.M. (1997). Prostaglandins and cancer. Front. Biosci..

[12] Hirsh P.D., Campbell W.B., Willerson J.T., Hillis L. (1981). Prostaglandins and ischemic heart disease. Am. J. Med..

[13] Pitt B., Shea M.J., Romson J.L., Lucchesi B.R. (1983). Prostaglandins and Prostaglandin Inhibitors in Ischemic Heart Disease. Ann. Intern. Med..

[14] O’Brien W.F. (1995). The Role of Prostaglandins in Labor and Delivery. Clin. Perinatol..

[15] Chwalisz K. (1994). The use of progesterone antagonists for cervical ripening and as an adjunct to labour and delivery. Hum. Reprod..

[16] Yount S.M., Lassiter N. (2013). The Pharmacology of Prostaglandins for Induction of Labor. J. Midwifery Women’s Health.

[17] Stoff J.S. (1986). Prostaglandins and hypertension. Am. J. Med..

[18] Smith M.C., Dunn M.J. (1985). The Role of Prostaglandins in Human Hypertension. Am. J. Kidney Dis..

[19] Peebles R.S. (2019). Prostaglandins in asthma and allergic diseases. Pharmacol. Ther..

[20] Antonova M., Wienecke T., Olesen J., Ashina M. (2013). Prostaglandins in migraine. Curr. Opin. Neurol..

[21] Hayaishi O., Matsumura H. (1995). Prostaglandins and sleep. Adv. Neuroimmunol..

[22] Kawaguchi H., Pilbeam C.C., Harrison J.R., Raisz L.G. (1995). The role of prostaglandins in the regulation of bone metabolism. Clin. Orthop. Relat. Res..

[23] Miller S.C., Marks S.C. (1994). Effects of prostaglandins on the skeleton. Clin. Plast. Surg..

[24] Akaogi J., Nozaki T., Satoh M., Yamada H. (2006). Role of PGE2 and EP receptors in the pathogenesis of rheumatoid arthritis and as a novel therapeutic strategy. Endocr. Metab. Immune Disord. Drug Targets.

[25] Samuelsson B., Folco G., Granström E., Kindahl H., Malmsten C. (1978). Prostaglandins and thromboxanes: Biochemical and physiological considerations. Adv. Prostaglandin Thromboxane Res..

[26] Park J.Y., Pillinger M.H., Abramson S.B. (2006). Prostaglandin E2 synthesis and secretion: The role of PGE2 synthases. Clin. Immunol..

[27] Serhan C.N., Levy B. (2003). Success of prostaglandin E2 in structure-function is a challenge for structure-based therapeutics. Proc. Natl. Acad. Sci. USA.

[28] Backlund M.G., Mann J.R., DuBois R.N. (2005). Mechanisms for the Prevention of Gastrointestinal Cancer: The Role of Prostaglandin E2. Oncology.

[29] Sugimoto Y., Narumiya S. (2007). Prostaglandin E Receptors. J. Biol. Chem..

[30] Nielsen M.S., Axelsen L.N., Sorgen P.L., Verma V., Delmar M., Holstein-Rathlou N.-H. (2012). Gap Junctions. Compr. Physiol..

[31] Maeda S., Tsukihara T. (2011). Structure of the gap junction channel and its implications for its biological functions. Cell. Mol. Life Sci..

[32] Harris A.L. (2007). Connexin channel permeability to cytoplasmic molecules. Prog. Biophys. Mol. Biol..

[33] Ek-Vitorín J.F., Burt J.M. (2013). Structural basis for the selective permeability of channels made of communicating junction proteins. Biochim. Biophys. Acta BBA Biomembr..

[34] Hervé J.-C., Derangeon M. (2012). Gap-junction-mediated cell-to-cell communication. Cell Tissue Res..

[35] Ekurtenbach S., Ekurtenbach S., Ezoidl G. (2014). Gap junction modulation and its implications for heart function. Front. Physiol..

[36] Desplantez T., Dupont E., Severs N.J., Weingart R. (2007). Gap Junction Channels and Cardiac Impulse Propagation. J. Membr. Biol..

[37] Brink P.R. (1998). Gap junctions in vascular smooth muscle. Acta Physiol. Scand..

[38] Daniel E.E., Wang Y.-F. (1999). Gap junctions in intestinal smooth muscle and interstitial cells of Cajal. Microsc. Res. Tech..

[39] Belousov A.B., Fontes J.D. (2014). Neuronal gap junction coupling as the primary determinant of the extent of glutamate-mediated excitotoxicity. J. Neural Transm..

[40] Belousov A.B. (2012). The regulation and role of neuronal gap junctions during neuronal injury. Channels.

[41] Meda P. (2018). Gap junction proteins are key drivers of endocrine function. Biochim. Biophys. Acta BBA Biomembr..

[42] Serre-Beinier V., Mas C., Calabrese A., Caton D., Bauquis J., Caille R., Charollais A., Cirulli V., Meda P. (2002). Connexins and secretion. Biol. Cell.

[43] Koval M. (2002). Sharing signals: Connecting lung epithelial cells with gap junction channels. Am. J. Physiol. Cell. Mol. Physiol..

[44] Garcia-Vega L., O’Shaughnessy E., Albuloushi A., Martin P. (2021). Connexins and the Epithelial Tissue Barrier: A Focus on Connexin 26. Biology.

[45] Radu A., Dahl G., Loewenstein W.R. (1982). Hormonal regulation of cell junction permeability: Upregulation by catecholamine and prostaglandin E1. J. Membr. Biol..

[46] Gregory M., Cyr D.G. (2018). Effects of prostaglandin E2 on gap junction protein alpha 1 in the rat epididymis. Biol. Reprod..

[47] Garfield R.E., Kannan M.S., Daniel E.E. (1980). Gap junction formation in myometrium: Control by estrogens, progesterone, and prostaglandins. Am. J. Physiol. Physiol..

[48] Cheng B., Kato Y., Zhao S., Luo J., Sprague E., Bonewald L.F., Jiang J.X. (2001). PGE2 Is Essential for Gap Junction-Mediated Intercellular Communication between Osteocyte-Like MLO-Y4 Cells in Response to Mechanical Strain. Endocrinology.

[49] Bostanci M.O., Bağirici F. (2006). The effects of octanol on penicillin induced epileptiform activity in rats: An in vivo study. Epilepsy Res..

[50] Manjarrez-Marmolejo J., Franco-Pérez J. (2016). Gap Junction Blockers: An Overview of their Effects on Induced Seizures in Animal Models. Curr. Neuropharmacol..

[51] Marandykina A., Palacios-Prado N., Rimkutė L., Skeberdis V.A., Bukauskas F.F. (2013). Regulation of connexin36 gap junction channels by n-alkanols and arachidonic acid. J. Physiol..

[52] Pan F., Mills S.L., Massey S.C. (2007). Screening of gap junction antagonists on dye coupling in the rabbit retina. Vis. Neurosci..

[53] Srinivas M., Hopperstad M.G., Spray D.C. (2001). Quinine blocks specific gap junction channel subtypes. Proc. Natl. Acad. Sci. USA.

[54] Paramanathan T., Vladescu I., McCauley M.J., Rouzina I., Williams M.C. (2012). Force spectroscopy reveals the DNA structural dynamics that govern the slow binding of Actinomycin, D. Nucleic Acids Res..

[55] Sobell H.M. (1985). Actinomycin and DNA transcription. Proc. Natl. Acad. Sci. USA.

[56] Choong M.L., Yang H., Lee M.A., Lane D.P. (2009). Specific activation of the p53 pathway by low dose actinomycin D: A new route to p53 based cyclotherapy. Cell Cycle.

[57] Ratnadiwakara M., Änkö M.-L. (2018). mRNA Stability Assay Using Transcription Inhibition by Actinomycin D in Mouse Pluripotent Stem Cells. Bio-Protocol.

[58] Obrig T.G., Culp W.J., McKeehan W.L., Hardesty B. (1971). The mechanism by which cy-cloheximide and related glutarimide antibiotics inhibit peptide synthesis on re-ticulocyte ribosomes. J. Biol. Chem..

[59] Lee S., Liu B., Lee S., Huang S.-X., Shen B., Qian S.-B. (2012). Global mapping of translation initiation sites in mam-malian cells at single-nucleotide resolution. Proc. Natl. Acad. Sci. USA.

[60] Jiang X., Coffino P., Li X. (2004). Development of a method for screening short-lived proteins using green fluorescent protein. Genome Biol..

[61] Hardesty B., Obrig T., Irvin J., Culp W. (1973). The Effect of Sodium Fluoride, Edeine, and Cycloheximide on Peptide Synthesis with Reticulocyte Ribosomes. Gene Expr. Regul..

[62] Fujiwara T., Oda K., Yokota S., Takatsuki A., Ikehara Y. (1988). Brefeldin A causes disassembly of the Golgi complex and accumulation of secretory proteins in the endoplasmic reticulum. J. Biol. Chem..

[63] Ayala J. (1994). Transport and internal organization of membranes: Vesicles, membrane networks and GTP-binding proteins. J. Cell Sci..

[64] Thyberg J., Moskalewski S. (1999). Role of Microtubules in the Organization of the Golgi Complex. Exp. Cell Res..

[65] Mejillano M.R., Shivanna B.D., Himes R.H. (1996). Studies on the nocodazo-le-induced GTPase activity of tubulin. Arch. Biochem. Biophys..

[66] Xi M., Gerriets V. (2020). Prostaglandin E2 (Dinoprostone). 2020 Jun 22. StatPearls [Internet].

[67] Toh H., Ichikawa A., Narumiya S. (1995). Molecular evolution of receptors for eicosanoids. FEBS Lett..

[68] Markovič T., Jakopin Ž., Dolenc M.S., Mlinarič-Raščan I. (2017). Structural features of subtype-selective EP receptor modulators. Drug Discov. Today.

[69] Abramovitz M., Adam M., Boie Y., Carrière M.-C., Denis D., Godbout C., Lamontagne S., Rochette C., Sawyer N., Tremblay N.M. (2000). The utilization of recombinant prostanoid receptors to determine the affinities and selectivities of prostaglandins and related analogs. Biochim. Biophys. Acta BBA Mol. Cell Biol. Lipids.

[70] Juteau H., Gareau Y., Labelle M., Sturino C.F., Sawyer N., Tremblay N., Lamontagne S., Carrière M.-C., Denis D., Metters K.M. (2001). Structure–activity relationship of cinnamic acylsulfonamide analogues on the human EP3 prostanoid receptor. Bioorg. Med. Chem..

[71] Jiang J., Ganesh T., Du Y., Quan Y., Serrano G., Qui M., Speigel I., Rojas A., Lelutiu N., Dingledine R. (2012). Small molecule antagonist reveals seizu-re-induced mediation of neuronal injury by prostaglandin E2 receptor subtype EP2. Proc. Natl. Acad. Sci. USA.

[72] Jiang J., Dingledine R. (2012). Role of Prostaglandin Receptor EP2 in the Regulations of Cancer Cell Proliferation, Invasion, and Inflammation. J. Pharmacol. Exp. Ther..

[73] Forselles K.J.A., Root J., Clarke T., Davey D., Aughton K., Dack K., Pullen N. (2011). In vitro and in vivo characterization of PF-04418948, a novel, potent and selective prostaglandin EP2 receptor antagonist. Br. J. Pharmacol..

[74] Kiriyama M., Ushikubi F., Kobayashi T., Hirata M., Sugimoto Y., Narumiya S. (1997). Ligand binding specificities of the eight types and subtypes of the mouse prostanoid re-ceptors ex-pressed in Chinese hamster ovary cells. Br. J. Pharmacol..

[75] Lawrence R.A., Jones R.L. (1992). Investigation of the prostaglandin E (EP-) receptor subtype mediating relaxation of the rabbit jugular vein. Br. J. Pharmacol..

[76] Tani K., Naganawa A., Ishida A., Egashira H., Sagawa K., Harada H., Ogawa M., Maruyama T., Ohuchida S., Nakai H. (2001). Design and Synthesis of a Highly Selective EP2-Receptor Agonist. Bioorganic Med. Chem. Lett..

[77] Woodward D.F., Jones R.L., Narumiya S. (2011). International Union of Basic and Clinical Pharmacology. LXXXIII: Classification of Prostanoid Receptors, Updating 15 Years of Progress. Pharmacol. Rev..

[78] Haslam R.J., Davidson M.M.L., Desjardins J.V. (1978). Inhibition of adenylate cyclase by adenosine analogues in preparations of broken and intact human platelets. Evidence for the unidirectional control of platelet function by cyclic AMP. Biochem. J..

[79] Davies S.P., Reddy H., Caivano M., Cohen P. (2000). Specificity and mechanism of action of some commonly used protein kinase inhibitors. Biochem. J..

[80] Engh R.A., Girod A., Kinzel V., Huber R., Bossemeyer D. (1996). Crystal Structures of Catalytic Subunit of cAMP-dependent Protein Kinase in Complex with Isoquinolinesulfonyl Protein Kinase Inhibitors H7, H8, and H89. J. Biol. Chem..

[81] Kurzrok R., Lieb C.C. (1930). Biochemical Studies of Human Semen. II. The Action of Semen on the Human Uterus. Exp. Biol. Med..

[82] Von Euler U. (1983). History and development of prostaglandins. Gen. Pharmacol. Vasc. Syst..

[83] Goldblatt M.W. (1935). Properties of human seminal plasma. J. Physiol..

[84] Wilson D.E. (1974). The Medical Significance of Prostaglandins. Arch. Intern. Med..

[85] Miller S.B. (2006). Prostaglandins in Health and Disease: An Overview. Semin. Arthritis Rheum..

[86] Rao P., Knaus E.E. (2008). Evolution of Nonsteroidal Anti-Inflammatory Drugs (NSAIDs): Cyclooxygenase (COX) Inhibition and Beyond. J. Pharm. Pharm. Sci..

[87] Rainsford K. (2007). Anti-Inflammatory Drugs in the 21st Century. Alzheimer’s Dis..

[88] Bertolini A., Ottani A., Sandrini M. (2002). Selective COX-2 Inhibitors and Dual Acting Anti-inflammatory Drugs: Critical Remarks. Curr. Med. Chem..

[89] Cereijido M., Ehrenfeld J., Meza I., Martínez-Palomo A. (1980). Structural and functional membrane polarity in cultured monolayers of MDCK cells. J. Membr. Biol..

[90] Cereijido M., Stefani E., Palomo A.M. (1980). Occluding junctions in a cultured transporting epithelium: Structural and functional heterogeneity. J. Membr. Biol..

[91] Cereijido M., González-Mariscal L., Borboa L. (1983). Occluding junctions and paracellular pathways studied in monolayers of MDCK cells. J. Exp. Biol..

[92] Meza I., Ibarra G., Sabanero M., Martinez-Palomo A., Cereijido M. (1980). Occluding junctions and cytoskeletal components in a cultured transporting epithelium. J. Cell Biol..

[93] Dukes J.D., Whitley P., Chalmers A.D. (2011). The MDCK variety pack: Choosing the right strain. BMC Cell Biol..

[94] Abbaci M., Barberi-Heyob M., Blondel W., Guillemin F., Didelon J. (2008). Advantages and limitations of commonly used methods to assay the molecular permeability of gap junctional intercellular communication. Biotechniques.

[95] El-Fouly M.H., Trosko J.E., Chang C.-C. (1987). Scrape-loading and dye transfer. Exp. Cell Res..

[96] Babica P., Sovadinová I., Upham B.L. (2016). Scrape Loading/Dye Transfer Assay. Methods Mol. Biol..

[97] Larre I., Ponce A., Fiorentino R., Shoshani L., Contreras R.G., Cereijido M. (2006). Contacts and cooperation between cells depend on the hormone ouabain. Proc. Natl. Acad. Sci. USA.

[98] Ponce A., Larre I., Castillo A., Garcia-Villegas R., Romero A., Flores-Maldonado C., Martinez-Rendón J., Contreras R.G., Cereijido M. (2014). Ouabain Increases Gap Junctional Communication in Epithelial Cells. Cell. Physiol. Biochem..

[99] Del Toro A.O., Jimenez L., Hinojosa L., Martínez-Rendón J., Castillo A., Cereijido M., Ponce A. (2019). Influence of Endogenous Cardiac Glycosides, Digoxin, and Marinobufagenin in the Physiology of Epithelial Cells. Cardiol. Res. Pr..

[100] Matlhagela K., Taub M. (2006). Involvement of EP1 and EP2 receptors in the regulation of the Na,K-ATPase by prostaglandins in MDCK cells. Prostaglandins Other Lipid Mediat..

[101] Regan J.W. (2003). EP2 and EP4 prostanoid receptor signaling. Life Sci..

[102] Ponce A., Larre I., Castillo A., Flores-Maldonado C., Verdejo-Torres O., Contreras R.G., Cereijido M. (2016). Ouabain Modulates the Distribution of Connexin 43 in Epithelial Cells. Cell. Physiol. Biochem..

[103] Shen V., Rifas L., Kohler G., Peck W.A. (2009). Prostaglandins change cell shape and increase intercellular gap junctions in osteoblasts cultured from rat fetal calvaria. J. Bone Miner. Res..

[104] Schlemmer S.R., Kaufman D.G. (2012). Re-establishment of gap junctional intercellular communication (GJIC) between human endometrial carcinomas by prostaglandin E2. Exp. Mol. Pathol..

[105] Taub M., Parker R., Mathivanan P., Ariff M.A.M., Rudra T. (2014). Antagonism of the prostaglandin E2 EP1 receptor in MDCK cells increases growth through activation of Akt and the epidermal growth factor receptor. Am. J. Physiol. Physiol..

[106] Karpisheh V., Nikkhoo A., Hojjat-Farsangi M., Namdar A., Azizi G., Ghalamfarsa G., Sabz G., Yousefi M., Yousefi B., Jadidi-Niaragh F. (2019). Prostaglandin E2 as a potent therapeutic target for treatment of colon cancer. Prostaglandins Other Lipid Mediat..

